# CD44 Glycosylation as a Therapeutic Target in Oncology

**DOI:** 10.3389/fonc.2022.883831

**Published:** 2022-07-21

**Authors:** Chengcheng Liao, Qian Wang, Jiaxing An, Jie Chen, Xiaolan Li, Qian Long, Linlin Xiao, Xiaoyan Guan, Jianguo Liu

**Affiliations:** ^1^ Department of Orthodontics II, Affiliated Stomatological Hospital of Zunyi Medical University, Zunyi, China; ^2^ Oral Disease Research Key Laboratory of Guizhou Tertiary Institution, School of Stomatology, Zunyi Medical University, Zunyi, China; ^3^ Microbial Resources and Drug Development Key Laboratory of Guizhou Tertiary Institution, Life Sciences Institute, Zunyi Medical University, Zunyi, China; ^4^ Department of Gastroenterology, Affiliated Hospital of Zunyi Medical University, Zunyi, China; ^5^ Department of Urology, The Third Affiliated Hospital of Zunyi Medical University, Zunyi, China

**Keywords:** CD44, glycosylation, hyaluronic acid, fucosylation, sialylation, HCELL

## Abstract

The interaction of non-kinase transmembrane glycoprotein CD44 with ligands including hyaluronic acid (HA) is closely related to the occurrence and development of tumors. Changes in CD44 glycosylation can regulate its binding to HA, Siglec-15, fibronectin, TM4SF5, PRG4, FGF2, collagen and podoplanin and activate or inhibit c-Src/STAT3/Twist1/Bmi1, PI3K/AKT/mTOR, ERK/NF-κB/NANOG and other signaling pathways, thereby having a profound impact on the tumor microenvironment and tumor cell fate. However, the glycosylation of CD44 is complex and largely unknown, and the current understanding of how CD44 glycosylation affects tumors is limited. These issues must be addressed before targeted CD44 glycosylation can be applied to treat human cancers.

## Introduction

CD44 is a nonkinase family and single-transmembrane glycoprotein that is expressed at different levels on the cell membranes of embryonic stem cells, bone marrow cells, tumor cells, etc. (1). In humans, CD44 is encoded by 19 exons, 10 of which are constant across all subtypes. The canonical forms of CD44 (CD44s) are encoded by 10 constant exons. CD44 variant isoforms (CD44v1-10) are produced by alternative splicing with any combination of 10 constant exons and the remaining 9 variant exons (2, 3). CD44s and various CD44v isoforms have overlapping and distinct functional roles. CD44v isoforms have additional binding motifs that facilitate CD44 interactions with molecules in the microenvironment (4). CD44v isoforms can act as coreceptors by binding/sequestering growth factors on the cell surface and presenting these growth factors to their specific receptors (5). CD44 promotes the stemness of cancer stem cells through interactions with HA, extracellular matrix components, growth factors, and cytokines (1). CD44 has been identified as a surface marker of cancer stem cell (CSC), especially the CD44v subtype is widely used to isolate and enrich CSC in different types of cancers (6). The CD44 transmembrane glycoprotein family not only establishes specific transmembrane complexes but also organizes signaling cascades through association with the actin cytoskeleton (7). Thus, CD44 is a signaling platform that integrates cellular microenvironmental signals, growth factor and cytokine signals and transduces signals to membrane-associated cytoskeletal proteins or the nucleus to regulate cell-matrix adhesion, cell migration, proliferation, differentiation and survival (6). Therefore, targeting different CD44 variants may be a promising therapeutic target for malignancies.

As a common feature of cancers, aberrant glycosylation is involved in fundamental molecular and cellular biological processes in cancer such as cell signaling and communication, tumor cell division and invasion, cell-matrix interactions, tumor angiogenesis, immune regulation, and metastasis formation (8). A growing body of biochemical, molecular and genetic studies suggests that alterations in protein glycosylation may be a major contributor to the tumorigenic transformation process, with significant effects on tumor disease progression (9). In this review, we describe the glycosylation modification characteristics and the potential of glycosylation alterations as a tumor therapy involving the glycoprotein CD44.

## The Structure and Glycosylation Domain of CD44

**Figure 1:**
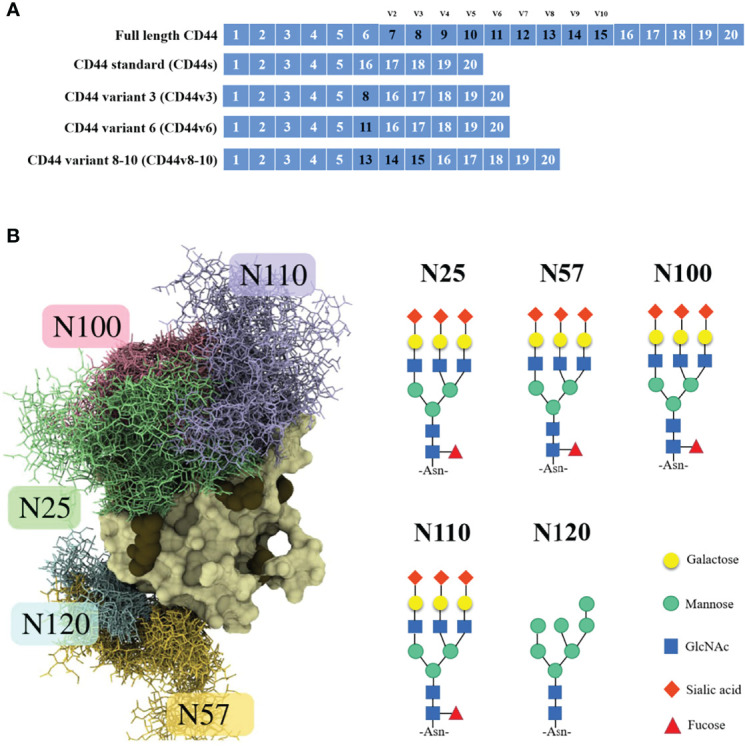
*CD44* gene and CD44s protein structure. **(A)** Schematic representation of full-length *CD44*, *CD44s*, *CD44v3*, *CD44v6* and *CD44v8-10.* **(B)** Example structure and N-glycan pattern of myeloma CD44-HABD monosialo (12).

The CD44 protein has four main structures: an extracellular region, a stem region (standard stem region and/or variable stem region), a transmembrane region (TM) and a short C-terminal intracellular/cytoplasmic region (CP) (10). The extracellular part consists of 7 extracellular domains (constant exons 1, 5, 6, 7), including the N-terminal domain (ligand binding region). The stem region (alternatively spliced region) inserts one or more variant exons between exon 5 and exon 6. The transmembrane region is encoded by exon 8, whereas the cytoplasmic region is encoded by exons 9 or 10. However, exon 9 is spliced out in almost all CD44 cDNA isoforms (11). CD44v has different structures in the stem region (**Figure 1A**), resulting in different functions (10). Due to the attachment of side chains, the conserved form of CD44 (37 kDa) expands to 80-100 kDa, with some isoforms exceeding 200 kDa due to the high degree of glycosylation (11). The extracellular domain of the CD44 protein is also known as the extracellular HA-binding domain (HABD) (13). CD44-HABD mainly binds to hyaluronic acid (HA), collagen, laminin and fibronectin (14–16).

**Figure 2:**
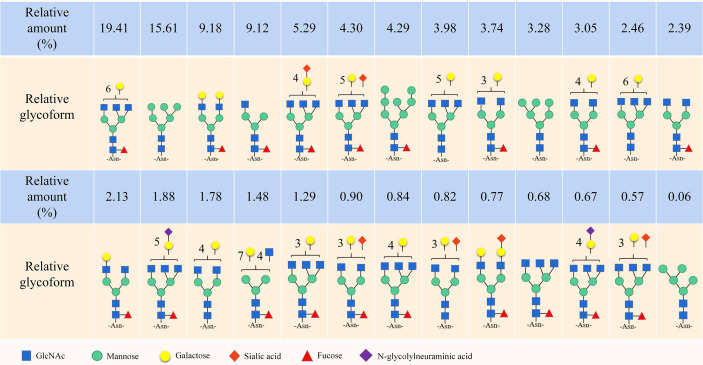
N-Glycans mudel presented on CD44s.

Notably, the CD44 extracellular structure is posttranslationally modified by N-glycans, O-glycans and glycosaminoglycans (heparan sulfate [HS], chondroitin sulfate [CS] and keratan sulfate) (17–19). Five conserved N-glycosylation consensus sites are located at amino-terminal 120 aa of CD44 (**Figure 1B**) (20). Mutation of any one of the five N-glycosylation sites of human CD44 results in the loss of wild-type CD44-mediated adhesion in active human cell lines (21). Nonsialylated and nonsucucosylated complex glycans dominate the N-glycans of CD44s (**Figure 2**) . In addition, the site-specific N-glycan profiles analyzed using LC-ESI-MS (E) showed that the vast majority of glycosyl groups, except for glycosyl N100, contained complex-type sugars, and high-mannose-type N-glycans occupied glycosyl N100 (22). The level of N-glycosylation may play a key role in CD44 activation-specific ligand binding (23–25). Most of the potential sites for O-glycosylation are located at the membrane proximal end of the CD44 ectodomain. A total of 146 O-glycan sites were predicted (26). Colon cancer cells modify CD44 with O-linked glycosyl groups, blocking CD44-mediated adhesion to HA (27). However, some studies suggest that the binding of HA to CD44 is not related to the level of CD44 O-glycosylation (25, 28).

Signals for glycosaminoglycan (GAG) assembly are encoded by the proteoglycan backbone. GAG synthesis occurs at serine followed by glycine, one or more proximal acidic amino acids. In addition, some acceptor sites are modified with CS only, and some acceptor sites are modified with both CS and HS (29). This suggests that CS synthesis occurs by default wherever GAGs can be attached, whereas HS assembly requires an additional signal. Duplication of the SG motif and signals found in the proteoglycan backbone also include proximal hydrophobic residues, when HS is assembled (30–32). The SGSG motif in the exon of CD44v3 is the only assembly site for HS, and the HS and CS linking at this site is abolished by serine to alanine (AGAG) mutation in the V3 motif. Eight amino acids downstream of the SGSG site in the V3 region are responsible for the specific addition of HS to this site (29). The E5 exon is the only exon in CD44 that supports GAG assembly and is modified by CS. If the 8 amino acids downstream of the first SG site of the CD44 E5 exon are exchanged with the 8 amino acids downstream of the SGSG site of the V3 exon, the SG site of E5 is modified by HS and CS. The 8 amino acids downstream of the first SG found in E5 are located downstream of SGSG in V3, and this site is modified by CS but not HS (29).

## N-Glycosylation and Sialylation Levels Regulate CD44 Binding to HA

HA is a group of polysaccharides, originally designated acid mucopolysaccharides, which are now labeled as glycosaminoglycans and are usually found in the connective tissue of vertebrates (33). Although HA is similar to other glycosaminoglycans, it consists of a single polysaccharide chain due to its molecular weight, which is in the millions of daltons (34). HA is composed of repeating disaccharide units of N-acetyl-D-glucosamine and D-glucuronic acid and is abundantly present in the extracellular matrix (35). HA is a polyanion that can self-associate or bind to water molecules, creating a viscous, gel-like environment when it is not bound to other molecules (35). The size of the HA polymer ranges from 50 to 20 million Da, and the function of HA is largely determined by its size (36). High molecular weight HA is present in normal, intact, healthy tissues and helps maintain normal homeostasis by inhibiting cell proliferation, migration, angiogenesis, inflammation and immunogenicity (37). Low-molecular-weight HA regulates tumor cell motility by binding to the CD44 receptor (38, 39). The interaction between HA and the CD44 receptor affects cell proliferation, survival, motility, invasiveness and chemoresistance (40). Posttranslational modifications, including glycosylation, sulfation, phosphorylation and clustering can all regulate the binding capacity of CD44 to HA (38, 41).

CD44 N-glycosylation regulates the "on" or "off" status of CD44 binding to HA (12, 24). Of the five N-glycosylation sites in CD44-HABD, Asn25 and Asn120 are required for HA binding and subsequent biological functions (20, 42). The negatively charged sugar chain attached to Asn25 directly hinders the binding of CD44 to HA (12), and N25 glycans can interact closely with nearby N100 and N110 glycans to form a sugar shield covering the typical binding sites of HA (43). Asn120 is located on the backside of the HABD close to Arg29 and does not appear to directly hinder the binding of CD44 to HA (12). A possible explanation is that charged glycans attached to Asn120 interfere with CD44 self-association and optimal binding of cross-linked CD44 molecules by directing them to adjacent sites on the same HA molecule (44). Furthermore, two mutations (IRAWB14 and IRAWB26) immediately adjacent to Asn120 in the CD44 structure, centered on Lys68, affect CD44 binding to HA (45).

The CD44 antigen is modified with sialic acids at the terminus of its glycans (46). The sialidase inhibitor 2-deoxy-2,3-dehydro-N-acetylneuraminic acid blocks HA recognition (47). When CD44 N-glycans are modified with sialic acid, sialic acid forms a competing intramolecular contact with the arginine side chain, thereby hindering the binding of HA (48). When CD44 N-glycans are modified with sialic acid, sialic acid forms a competing intramolecular contact with the arginine side chain, thereby hindering the binding of HA (48). Sialidase catalyzes the removal of sialic acid (49), including NEU1, NEU2, NEU3 and NEU4 and is associated with apoptosis, neuronal differentiation, and tumorigenesis (50). After NEU4 overexpression or activation, the modification of α-(2,3)-sialylation on CD44 decreases, and the binding affinity of CD44 for HA increases in HCC (51). Similarly, in airway inflammation, CD44 HA binding activity is also dependent on Neu1 enzymatic activity (52). The sialylation status of CD44 may be more important than its degree of expression for binding to HA (53).

N-glycosylation at specific sites of CD44 and the level of sialylation modification on N-glycans together affect HA binding. The extent of primary CD44 glycosylation and the size of the attached oligosaccharides determine the coverage of the binding site to HA. It is worth noting that the addition of sialic acid had little effect on binding site coverage compared with similarly sized nonsialylated N-glycans (43). The inhibition of HA binding by sialylation may be due to the increased degree of negative charge. Smaller N-glycan types, such as simple GlcNAc residues, lack the range and charge necessary to negatively affect HA binding. Conversely, the presence of GlcNAc residues provides HA with an additional binding surface, potentially facilitating HA recognition by providing additional polar interaction sites and minimal binding barriers (43, 54). In addition, N-glycosylation of CD44-HABD promotes a secondary, less shielded but weaker (>10 μM) HA binding site (12, 55). In the case of CD44 N-glycosylation, the flanking arginines (R150, R154 and R162) are relatively more likely to interact with HA (43). Modulation of glycosylation alone cannot completely block CD44 binding to HA; however, this binding is weak enough that modulation of the CD44 N-glycan/HA axis remains a potential therapeutic target.

## CD44 O-Glycosylation Regulates The Aggressiveness of Cancer

The O-glycosylation process refers to the addition of N-acetylgalactosamine (GalNAc) to serine or threonine residues in proteins and the addition of other sugar branches to create more complex structures (56, 57). The biosynthesis of O-glycans is controlled by T-synthase (C1GalT1) (58, 59). The endoplasmic reticulum-localized chaperone Cosmc is required for C1GalT1 activity and expression (60, 61). Cosmc dysfunction results in inactive C1GalT1 and subsequent expression of the Tn antigen (62). Cosmc deficiency disrupts the CD44 O-glycosylation structure and subsequent inhibition of MAPK signaling leads to the inhibition of breast cancer cell growth *in vivo* (63). Deficiency or overexpression of Tn antigens occurs in many types of cancer, including gastric, colon, breast, lung, esophageal, prostate and endometrial cancers (64), which suggests that aberrant O-glycosylation occurs frequently during tumor development. In the complex O-glycosylation process, the formation of the Core 1 O-glycosyl is considered to be one of the main modes of glycosylation. Core 1-mediated disruption of CD44 O-glycosylation allows truncated CD44 in human colon cancer cell lines to be secreted into the extracellular environment *via* microvesicles; thus, exosome CD44 may be a potential vehicle for aberrant O-glycosylation (65). In addition, higher levels of serum CD44 protein containing the STn structure have been shown to distinguish gastric cancer patients from healthy subjects (66, 67).

C1GalT1 is overexpressed in many cancers of epithelial origin, including colon, breast, gastric, HNSCC, esophagus, prostate, and hepatocellular carcinoma. C1GalT1 overexpression is also frequently associated with poorer prognosis and poorer patient survival (68). The oncogenic effects of C1GalT1 may be achieved by altering the glycosylation and function of receptor tyrosine kinases, cell surface integrins, and cell surface death receptors (68). However, human pancreatic cancer cells with knockout of C1GALT1 had an increased tendency for tumorigenesis and metastasis (69). By localizing Tn-containing glycoproteins in C1GalT1 KO cells, Leon et al. (70) found that Tn was significantly enriched on the CSC glycoprotein CD44. C1GalT1-mediated truncation of O-glycans on CD44 inactivates the ERK/NF-κB signaling pathway, resulting in NANOG expression in pancreatic cancer (PC) cells and changes in tumor stem cell characteristics. However, high C1GALT1 expression is associated with poor survival in pancreatic duct adenocarcinoma patients (71). Why does truncation of CD44 O-glycosylation contribute to downstream signaling? We speculate that excessive O-glycosylation, similar to incomplete O-glycosylation of CD44, affects the efficiency of CD44 function (68–71). Furthermore, the increased tumorigenicity of pancreatic cancer caused by knockdown of C1GalT1 may be also related to the truncation of o-glycosylation on MUC16 (69).

Proteoglycan 4 (PRG4) is a mucin-like glycoprotein originally found in synovial fluid, a secreted product of the intimal cells of joint tissue and present on the surface of articular cartilage (72). Extensive O-glycosylated mucin-like domains are required for the boundary lubrication and lytic properties of PRG4 at various biological interfaces *in vivo*, including articular cartilage, tendon, pericardium, and the ocular surface (72). PRG4 is a ligand for CD44 (73–75). Removal of CD44 O-glycosylation significantly increases rhPRG4 binding to CD44 (76). PRG4 has been observed to inhibit cancer progression through the CD44/TGF-β pathway (77, 78). However, it is unknown whether PRG4 inhibits tumor progression by binding to CD44, thereby competing with HA or other ligands.

## Fucosyltransferase-Mediated CD44 Fucosylation Promotes Tumor Progression

Fucosylation comprises the attachment of a fucose residue to N-glycans, O-glycans and glycolipids, and is one of the most common modifications (79). All fucosylation reactions in cells are catalyzed by focusyltransferases (FUTs). To date, 13 FUTs—FUT1 to 11, protein O-focusyltransferase 1 (POFUT1) and POFUT—have been identified (80). After CD44 N-acetylglucosamine is modified by α-(1,3)-focusylation, it can further form a sialyl-Lewis X (sLeX) structure, which can effectively bind to E-selectin (81, 82). Cell adhesion mediated by selectin and its carbohydrate ligand sLeX plays an important role in cancer metastasis (83). CD44 fucosylation improves the stemness of mouse bone marrow mesenchymal stem cells (84–87). During chemotherapy in canine lymphosarcoma, Xiong et al. (88) found that the concentration of CD44 fucosylated protein decreased by more than 2-fold, which may be related to tumor cell adhesion and migration. Increasing some specific forms of CD44 fucosylation in tumors is also thought to promote tumor progression.

MicroRNA 29b is a tumor suppressor with important effects on cancer progression (89). Specificity protein 1 (Sp1) is a well-known member of a family of transcription factors that also includes Sp2, Sp3, and Sp4, which are involved in processes such as cell growth, differentiation, apoptosis, and carcinogenesis (90). Sp1 is one of the targets of miR-29b (91, 92). Liu et al. (93) found that miR-29b/Sp1 mediates the malignancy of leukemia stem cells by regulating FUT4-mediated CD44 fucosylation in acute myeloid leukemia. CD44/E-selectin-binding signaling upregulates intercellular adhesion molecule 1 (ICAM-1) expression on the cell surface *via* the PKCα/p38/Sp1 pathway, thereby promoting melanoma cell metastasis (94). The E-selectin ligand activity of CD44 is conferred by sialylation and fucosylation modification of CD44 N-glycans (95). The lncRNA HOX transcribed antisense RNA (HOTAIR) is elevated in broad-spectrum tumors and is associated with metastasis and poor prognosis (96). In human tumors, HOTAIR competitively binds to miR-326, thereby regulating miR-326 expression (97–99). The HOTAIR/miR-326 axis was shown to regulate FUT6 expression to promote fucosylation of CD44 in colorectal cancer, and fucosylated CD44 could activate the PI3K/AKT/mTOR pathway to promote tumor progression (100). FUT6 mediates cell surface α-(1,3)-fucosylation, induces sLeX expression, and converts CD44 to hematopoietic cell E-/L-selectin ligand (HCELL) (82). Both FUT4 and FUT6 can modify α-(1,3)fucosyl bond formation. In addition, transfection of α-(1,2)-focusyltransferase (FUT1/2) cDNA into colon cancer cell lines resulted in cell surface expression of CD44 variants carrying the amino acid sequence encoded by exon v6 (CD44v6), thereby promoting tumorigenesis progress (101–104). CD44v6 is thought to be expressed and to have increased tumorigenicity in various human tumors, including colorectal, and head and neck squamous cell carcinoma (HNSCC) (105–107).

The enzyme α-(1,6)-focusyltransferase (FUT8) is the only fucosyltransferase responsible for protein N-glycan core fucosylation (108). In the Golgi apparatus, FUT8 transfers an L-focal site from guanosine diphosphate (GDP-β-L-focus) (GDP-fuc) to the innermost GlcNAc of N-glycans to form an α-1,6 fucosyl bond (109, 110). FUT8 knockout homozygous mice experience early postnatal death, severe growth retardation, and emphysema-like changes in the lungs (111). FUT8 has an important stabilizing effect on N-glycan structure, which allows FUT8 to regulate key glycoproteins. FUT8-mediated core fucosylation plays an important role in regulating the biological functions of EGFR, TGFBR, E-cadherin, PD1/PD-L1 and α3β1 integrin (80). However, there is still no report on the effect of removing core fucosylation modification from the CD44 protein.

## CD44 N-Glycosylation Regulates its Binding to TM4SF5

Hepatic transmembrane 4 L six family member 5 (TM4SF5) is a membrane protein and a member of the tetrase protein family, with four transmembrane domains, a cytoplasmic N- and C-terminus, and an intracellular loop. TM4SF5 is N-glycosylated on residues N138 and N155 and palmitoylated on cysteine residues near the cytoplasmic boundary of the transmembrane domain (112). Similar to other tetraterpenoids, TM4SF5 has been shown to interact with a variety of membrane proteins and receptors on the cell membrane, resulting in TM4SF5-enriched microdomains (T5ERMs) (113). TM4SF5 has been shown to form protein–protein complexes with CD44 (114), CD133 (115), CD151 (116), epidermal growth factor receptor (EGFR) (117), insulin-like growth factor 1 receptor (IGF1R) (118) and integrin-α5 (119) on the cell surface and play roles in tumor cell migration and anticancer drug resistance. The TM4SF5/CD44 interaction activates the proto-oncogene tyrosine-protein kinase Src (c-Src)/signal transducer and activator of transcription 3 (STAT3)/Twist-related protein 1 (Twist1)/B-cell-specific Moloney murine leukemia virus integration site 1 (Bmi1) signaling pathway (120), and epithelial-mesenchymal transition (EMT) (111), which makes TM4SF5/CD44 a potential target for tumor-targeted therapy with coexpression of TM4SF5 and CD44. The interaction between TM4SF5 and CD44 and the activation of the c-Src/STAT3/Twist1/Bmi1 pathway occur through the N-glycosylation modification of the extracellular domain of TM4SF5 and CD44 (114). Targeted therapy of TM4SF5 or TM4SF5/CD44 interaction may effectively inhibit tumor progression.

## CD44 Terminal Sialic Acids on Tumors Regulate Their Binding to Siglec-15

Siglec-15 was originally identified as a member of the Siglec family with structural features of sialic acid-binding immunoglobulin-type lectins (121). Siglec15 is upregulated in many human cancers, and as an immunosuppressive molecule that plays a role in the tumor microenvironment (TME), it is mutually exclusive with PD-L1 and has potential implications in patients with anti-PD-1/PD-L1 resistance (122, 123). CD44 is a ligand for Siglec-15 (124). In the presence of CD44 N-glycan α-(2,6)-linked sialic acid modifications, Siglec-15 interacts with CD44 and mediates liver cancer progression and metastasis by preventing CD44 lysosome-mediated degradation (125).

The *ST3GAL4* gene encodes β-galactosidase α-(2,3) sialyltransferase 4 and is involved in the biosynthesis of tumor antigens sLeX and sulfo sLeX (126). MicroRNA 193b targets ST3GAL4 to regulate CD44 sialylation *via* the NF-κB pathway, thereby accelerating osteoarthritis progression (127). The α-(2,3)-sialyltransferases ST3GAL1 and ST3GAL4 are the main enzymes for the synthesis of Siglec7 and Siglec-9 in tumor cells. Both ST3GAL4 and Siglec-7/9 have been shown to play important roles in human tumors (128, 129). However, whether ST3GAL4 regulates CD44 sialylation and plays a role in tumors still needs to be confirmed. In conclusion, inhibition of tumor progression by modulating CD44 sialylation appears to be a viable option.

## HCELLs Are Major E-/L-Selectin Ligands in Tumors

E-selectin is a cytokine-activated cell adhesion molecule expressed on endothelial cells and plays an important role in the adhesion of inflammatory and metastatic cancer cells to endothelial cells (130). L-selectin is an L-type transmembrane glycoprotein and cell adhesion molecule expressed on most circulating leukocytes and plays a role in regulating monocyte protrusion during monocyte transendothelial migration (131). Hematopoietic cell E/L-selectin ligand (HCELL) is a specific glycated form of sialofucosylated CD44 that is characteristically expressed on human hematopoietic stem cells and is the most potent E-selectin and L-selectin expressed on human cell prime ligands (132). According to the current commonly used CD44 nomenclature, the activities of HCELL on standard and variant CD44 isoforms are designated as HCELLs and HCELLv, respectively (133). The two isoforms of HCELL differ from each other at the protein backbone and glycosylation levels. The two HCELL isoforms have significantly different molecular weights: HCELL migrates as a band of 90-100 kDa on SDS–PAGE gels, whereas the HCELLv of colon cancer cells typically has a molecular weight of approximately 150 kDa (134). Metastatic MDA-MB-231 breast cancer cells express high levels of the ~170 kDa HCELLv4 isoform (135). HCELLs have been shown to be present at high levels in human malignant hematopoietic cells, including neonatal acute myeloid leukemia (AML) cells and the AML-derived primitive human hematopoietic progenitor cell line KG1a (95, 132, 136). In contrast, malignant cells of solid cancers predominantly express the HCELLv subtype (132, 133, 137).

HCELLv is characterized by the presentation of sialofucosylated glycosyl groups on O-glycans of the CD44 subtype. Despite these differences, cleavage-based *in vitro* assays suggest that HCELLs and HCELLv are equally potent ligands for E- and L-selectin (81). Current evidence suggests that HCELL is a key E-selectin ligand in breast cancer (138) and a major E-/L-selectin ligand in colon cancer (139). Human bone marrow-derived mesenchymal stem cells migrate across endothelial cells without the need for chemokine signaling through a VLA-4/vcam-1-dependent "Step 2-bypass pathway" after forced targeted expression of HCELL (140, 141). The discovery of HCELL and its function as a bone marrow homing receptor may have enormous implications for the realization of inverted glycobiology in clinical medicine.

## CD44 Chondroitin Sulfate Modification Mediates CD44 Binding to Fibronectin and Collagen in Tumors

Chondroitin sulfate (CS) is a sulfated glycosaminoglycan (GAG) distributed on the cell surface and in the extracellular space. CS chains are covalently linked to a core protein called CS proteoglycan (CSPG), which mediates protein–protein interactions between cells and the extracellular matrix (ECM) by maintaining the physical structure of the tissue, supporting various biological functions of CSPG (142). CSPG interacts with multiple transmembrane proteins, including integrins and receptor tyrosine kinases, and modulates cell signaling related to tumor cell proliferation, invasion, migration, angiogenesis, and metastasis (143, 144). CS and keratin sulfate are found on CD44 in certain cell types (145, 146). The addition of this glycosaminoglycan has been shown to modify the function of CD44. The keratin sulfate side chain on CD44H in highly metastatic colon cancer cells significantly reduces HA binding (146). Keratin sulfate modification of CD44 regulates hyaluronic acid adhesion through its B-loop domain (146). Tumor necrosis factor alpha or lipopolysaccharide and interferon gamma (LPS/IFNγ) stimulation in mouse bone marrow-derived macrophages induces HA binding by downregulating the sulfation of CD44 by CS (147). Chondroitin sulfate-modified CD44 (110 kDa) in mouse melanoma promotes melanoma cell motility (not adhesion and spread) on type I collagen. Furthermore, the binding of CD44 to type I collagen was mediated by chondroitin sulfate by affinity chromatography and solid-phase binding assays (148). The nonspliced core protein of CD44 is glycosylated by chondroitin sulfate, which promotes the migration of fibroblasts to the fibrin clot and the migration of endothelial cells on the fibrin matrix. The attachment of CD44 to fibronectin requires chondroitin sulfate modification (16, 149). CD44 chondroitin sulfate modification may be a target for inhibiting tumor cell motility and metastasis.

## CD44 Heparan Sulfate Modification Mediates CD44 Binding to Growth Factors

HS proteoglycan is ubiquitously expressed on the surface of most animal cells and in the extracellular matrix, and its function mainly depends on the interaction of the HS side chain with various proteins such as cytokines, growth factors and their receptors, and it plays an important role in tumor progression (150). In HS polysaccharides, negatively charged sulfate and carboxylic acid groups are arranged in various domains and generated through tightly regulated biosynthetic reactions, with great potential for structural change (151). The CD44v3 isoform containing the HS attachment site is overexpressed on the tumor epithelium of colorectal adenomas and most carcinomas compared with normal colon (152). CD44v3 is modified by HS and has been shown to bind growth factors (153, 154), promoting the binding of CD44 molecules to the cytoskeleton in colon cancer (155). In colorectal cancer, the heparin-like and chondroitin sulfate B side chains of CD44 bind to the laminin A5G27 peptide (156, 157). The A5G27 peptide has excellent specificity for cancer cells overexpressing CD44v3 and CD44v6 and inhibits the migration and invasion of cancer cells (158). A5G27 shares considerable sequence homology (69%) with a sequence in fibroblast growth factor 2 (FGF2) that binds heparin and the FGF receptor, which is essential for central cavity formation in FGF2 (159). CD44 binding to FGF2 specifically increases FGF2-mediated proliferation, migration and survival of tumor and endothelial cells, thereby increasing tumor growth and metastasis (160, 161). The A5G27 peptide inhibits melanoma metastasis and angiogenesis by reducing the biological activity of FGF2 by blocking the binding of FGF2 to the HS side chain of CD44v3 (162). Modulation of CD44 heparan sulfate modification may be a promising antitumor therapy.

## CD44 Glycosylation Negatively Regulates Podoplanin-CD44 Binding in Squamous Cell Carcinoma

Podoplanin (PDPN) is a type I transmembrane mucin-like sialoglycoprotein (163). PDPN is highly expressed on lymphatic endothelial cells and used as a marker of lymphangiogenesis (164). Mice deficient in PDPN die soon after birth due to abnormalities in the lungs, heart, and lymphatic vasculature (165). PDPN increases tumor cell clonality, EMT, migration, invasion, metastasis and inflammation in tumors including glioma, squamous cell carcinoma, mesothelioma and melanoma and is considered a potential tumor biomarker and therapy target (166). Podoplanin molecules lack obvious enzymatic motifs, so they must exert their biological and pathological functions through protein–protein interactions. The C-type lectin-like receptor 2 (CLEC-2), ERM (ezrin, radixin, moesin) protein family members ezrin and moesin, CD9 tetraspanin, standard isoforms of CD44s and CD44s have been found in different cell types and environments in which PDPN interacts (167). In squamous cell carcinoma (SCC) cells, CD44 and PDPN colocalize on cell surface protrusions, and CD44 is required for PDPN to promote directional and sustained movement of epithelial cells (168). CD44v3-10 is the main variant isoform coexpressed by CD44s and PDPN in human SCC cell lines (169). The interaction of PDPN and CD44 is mediated by the transmembrane and cytoplasmic domains and is negatively regulated by glycosylation of the extracellular domain of CD44 (169). Inhibition of PDPN-mediated tumor progression by regulating CD44 glycosylation deserves further in-depth research.

## Conclusion and Prospect

The level of CD44 N-glycosylation and sialylation negatively regulates the binding to HA; however, whether O-glycosylation has a similar effect still needs further study. The functioning of CD44 generally involves combining with some membrane proteins or the extracellular matrix, and N-glycosylation and O-glycosylation on the extracellular domain of CD44 cover or constitute the corresponding binding site, thereby regulating the tumor microenvironment and intracellular signal transduction.

The development of glycosyl-based cancer neoantigens as cancer vaccines and targeted therapies may pave the way for more effective and specific tumor targeting (170). The monoclonal antibody F77 is highly specific for prostate cancer and can recognize the glycosylation structure of CD44v10 (171). Bivalent F77 can induce apoptosis in prostate cancer cells, particularly at 4°C (172), where F77 has a higher binding affinity to its antigen (173). Downregulation of *CD44* or *FUT1* genes significantly reduced F77-induced apoptosis in prostate cancer cell lines, suggesting that the binding site of F77 may require fucosylation modification (172). KMP1 is an IgG1 antibody that specifically binds EJ, BIU-87, and T24 bladder cancer cell lines and bladder cancer tissue but not Lovo, HeLa, K562, HepG2, Jurkat, 293, or HCV29 cell lines, human erythrocytes, human lymphocytes or normal bladder tissue (174). However, the effectiveness of CD44 as a therapeutic or diagnostic target has not been fully demonstrated in some other studies. In addition, polysaccharide-based biomaterials HA and CS have attracted great interest as tumor drug delivery systems due to their good biocompatibility with and targeting to CD44. The use of such drugs, combined with drugs targeting the CD44 glycosylation site, may be able to achieve better therapeutic effects (175).

The critical biological roles of CD44s and CD44v in tumors have been extensively studied; however, the glycosylation patterns of CD44s and CD44v in different tumors are unknown. How to engineer the glycosylation pattern of the CD44 extracellular domain to achieve antitumor effects still requires much effort. Notably, the CD44 glycosylation pattern as a precise diagnostic marker in different tumors has also not been reported. The identification of glycan epitopes by tumor subtype may have potential applications in patient treatment stratification. Furthermore, the prospects for creating CAR-T cells specific to CD44 glycosylation could be very interesting work. The complexity of the glycosylation process and the lack of specific methods to study it hinder related research progress. However, recent advances in new methods such as cellular glycoengineering and high-throughput screening (HTS) have opened new avenues of discovery (176), which helps to explore the effect of different glycosylation of specific amino acid residues on the binding of different ligands

## Author Contributions

XG, LX and JL contributed to the conception and design of the review. CL wrote the manuscript. QW, JA and QL validate the manuscript. XL and JC contributed to the visualization of CD44s glycosylation. All authors contributed to manuscript revision, read, and approved the submitted version.

## Funding

This study was supported by the National Key R&D Program (2016YFC1102800); Guizhou Province Medical Biomaterials R&D Talent Base (QianRenLingFa [2018] No. 3); the Sixth Talent Foundation in Guizhou province (rcjd2019-9); the Graduate Research Fund of Guizhou Province (Qian-Jiao-He YJSCXJH [2019]087); Zunyi Medical Biomaterials R&D and Innovative Talent Base (ZunWei [2019] No. 69); the Youth Science and Technology Talents Growth Project of Guizhou Education Department (Qian-Jiao-He KY ZI [2018]236); Zunyi City and Zunyi Medical University Unite Fund [QianShiKeHe HZ Zi (2022)393], Outstanding Young Talent Project of Zunyi Medical University (17zy-002) (F-801); The Project to Cultivate Young Scientific; Master Fund of Zunyi Medical University (S-81) and Technological Talents in Colleges and Universities of Guizhou Province (Qian Jiao He KY [2021] 215).

## Conflict of Interest

The authors declare that the research was conducted in the absence of any commercial or financial relationships that could be construed as a potential conflict of interest.

## Publisher’s Note

All claims expressed in this article are solely those of the authors and do not necessarily represent those of their affiliated organizations, or those of the publisher, the editors and the reviewers. Any product that may be evaluated in this article, or claim that may be made by its manufacturer, is not guaranteed or endorsed by the publisher.
